# The mechanisms of action of vaccines containing aluminum adjuvants: an *in vitro* vs *in vivo* paradigm

**DOI:** 10.1186/s40064-015-0972-0

**Published:** 2015-04-16

**Authors:** Tirth Raj Ghimire

**Affiliations:** Division of Veterinary and Primate Health, Global Primate Network, Kathmandu, Nepal; Department of Zoology, Birendra Multiple Campus, Tribhuvan University, Chitwan, Nepal

**Keywords:** Alum internalization, Depot, Antigen targeting, Inflammasome, Innate and adaptive

## Abstract

Adjuvants such as the aluminum compounds (alum) have been dominantly used in many vaccines due to their immunopotentiation and safety records since 1920s. However, how these mineral agents influence the immune response to vaccination remains elusive. Many hypotheses exist as to the mode of action of these adjuvants, such as depot formation, antigen (Ag) targeting, and the induction of inflammation. These hypotheses are based on many *in vitro* and few *in vivo* studies. Understanding how cells interact with adjuvants *in vivo* will be crucial to fully understanding the mechanisms of action of these adjuvants. Interestingly, how alum influences the target cell at both the cellular and molecular level, and the consequent innate and adaptive responses, will be critical in the rational design of effective vaccines against many diseases. Thus, in this review, mechanisms of action of alum have been discussed based on available *in vitro* vs *in vivo* evidences to date.

## 1. Vaccines

Since the age of Edward Jenner, vaccines have revolutionized public health worldwide, successfully saving the lives of millions of people from infectious diseases such as diphtheria, *Haemophilus influenzae* type b (Hib) infection, hepatitis B viral infection, tetanus, measles, mumps, neonatal tetanus, pertussis, pneumococcal infection, rubella, and serogroup C meningococcal infection (Rappuoli et al. 2002; WHO 2010b). It has been about 30 years since the World Health Organization (WHO) announced the complete control and eradication of smallpox, achieved through the widespread application of the smallpox vaccine (Bonanni and Santos 2011). With increasing vaccine coverage, the eradication of polio is also nearly complete (WHO 2010a, b). This can be explained by the 99% reduction in the number of polio cases since 1988, leaving only Nigeria, Pakistan, and Afghanistan as polio-endemic countries (WHO 2014) (http://www.who.int/mediacentre/factsheets/fs114/en/, Accessed on 4 February, 2015). Therefore, vaccine discovery has been one of the greatest achievements and one of the most economic and safe interventions of biomedical science.

While vaccines are one of the most successful scientific breakthroughs, the underlying immunology requires further research. The success of a vaccine depends on the quality, magnitude, and duration of the generated adaptive immune response following vaccination. To initiate an adaptive immune response, a number of signals are required by naïve T cells. Among these signals, signal 1 is the vaccine-derived, peptide antigen (Ag) bound to major histocompatibility (MHC) class II and class I displayed on the surface of antigen presenting cells (APCs) (Mueller et al. 1989; Watts 1997; Nelson et al. 1997). Signal 2 is also known as ‘costimulation’ and importantly, together with signal 1, induces immune response. Signal 2 involves cross-linking of CD28 and other receptors on the T cell by costimulatory molecules such as B7-1 (CD80), B7-2 (CD86), and other ligands expressed by the APC. Signal 3 is provided by cytokines and is delivered from the APC to the T cell that determines its differentiation into an effector cell. Both Signal 2 and signal 3 are provided to T cells by activated and matured APCs like dendritic cells (DCs). Mature DCs are able to induce T cell clonal expansion and prime immune responses (Reis e Sousa and Germain 1995; Reis e Sousa 2006) and are thus central to the understanding of vaccines.

**Figure 1:**
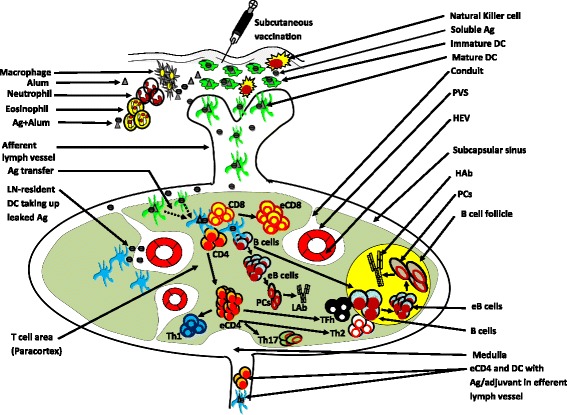
Current understanding of immunology of vaccines containing alum adjuvants *in vivo*. Alum immunization leads to recruitment of neutrophil, natural killer cell, macrophage, eosinophil, and immature DC at the injection site. Immature DCs take up soluble Ag released from alum or particulate Ags mixed in alum in the subcutaneous areas and migrate towards draining lymph node (DLN). Soluble Ag can reach to DLN without help of DCs. In T cell area (paracortex), soluble Ags leaked out of conduits are taken up by resident DCs. DCs present Ag to the naïve T cells or transfer Ag to the resident DCs that present Ag to those T cells. In addition, B cells are capable of binding to this Ag with their surface immunoglobulins. B cells undergo activation, produce effector B cells (eB cells), and rapidly differentiate in plasma cells (PCs). Plasma cells produce low-affinity antibodies (LAb). B cells also migrate to B cell follicle. On the other hand, as a result of CD8 and CD4 T cell activation, effector CD8 (eCD8) T cells and effector CD4 (eCD4) T cells are produced. The eCD4 polarizes into T helper (Th) 1, 2, 17 or T follicular helper (Tfh) cells though alum induces development of mostly Th2 and Tfh cells. Tfh or Th2 may reach to the border of B cell follicle to activate B cells that produce eB cells and then PCs. The PCs consequently produce and secrete high-affinity antibody (HAb). Few alum-fed DCs and eCD4 may travel to efferent lymph vessels to reach into the distant LN *in vivo*. Ag: antigen, HEV: high endothelial venule, PVS: perivenular space (see text for explanation).

DCs undergo maturation processes when they receive specific cues from their environment, such as exposure to toll-like receptor (TLR) ligands, necrosis, inflammatory soluble factors (cytokines), T cell ligands (such as CD40 ligands), and disruption of homotypic contacts between immature DCs (Reis e Sousa 2006; Trombetta and Mellman 2005; Sauter et al. 2000). DC maturation involves changes in both location and phenotype of DC, turning it from a cell specialized in surveillance into a potent activator of naïve T cell. DC maturation is characterized by the appearance of dendritic processes, the increased expression of MHCII molecules, costimulatory molecules, and chemokine receptor 7 (CCR7) (Yanagihara et al. 1998; Sallusto et al. 1999; Huang et al. 2000), and the production of cytokines. In this context, the MHCII molecules present Ag, costimulatory molecules contribute to activate the T cells, the CCR7 chemokine receptor mediates migration of the cells to the draining lymph node (DLN), and cytokines are involved in a variety of functions, e.g. cellular trafficking to vaccine-injected sites and DLNs, T cell activation, and T cell polarization (Figure 1).

In the LN, DCs located around the T cell entry site express Epstein-Barr virus induced receptor ligand chemokine (ELC) or chemokine ligand 9 (CCL19) and secondary lymphoid tissue chemokine (SLC) or CCL21 that enhance DC-T cell attraction. In the LN paracortex, B cells bind Ags with their surface immunoglobulins and become activated. As a result, they rapidly differentiate in plasma cells and produce low-affinity antibody (Ab). DC-T cell interaction in the paracortex leads to Ag-specific CD4^+^ T cell activation, expansion, and polarization into T helper (Th) 1, 2, and 17, and T follicular helper (Tfh) cells. Th2 and Tfh cells initiate germinal center reaction where these cells and follicular DCs (FDCs) provide strong activating signals to B cells. The activated B cells produce plasma cells that secrete Ag-specific high-affinity Abs (Figure 1).

The advancing field of biomedical science has increased the practical and theoretical understanding of pathogen biology, molecular biology, biochemistry, immunology, and biotechnology. Therefore, vaccine development has progressed from trial and error- based empirical vaccine studies towards more rational and reductionist approaches (Van Regenmortel 2004). Nevertheless, these approaches have had limited success in developing effective vaccines against emerging diseases like Human Immunodeficiency Virus/Acquired Immunodeficiency Syndrome (HIV/AIDS) and re-emerging diseases like tuberculosis (TB) and malaria. This may be due to a number of factors, such as rapid clearance from the body, poor recognition by the immune system, and failure to adequately stimulate appropriate immune cells (Edelman 2002; Edelman and Tacket 1990). Therefore, while appropriate and safe Ag discovery has been the main target of vaccinology, in recent years the development of adjuvants (a term derived from the Latin ‘to help’) that increase the immunogenicity of the Ags is also gaining equal importance. Adjuvants are used in vaccines to reduce the dose of vaccines, to induce the particular protective response (CD4 vs CD8 and Th1 vs Th2) and to enhance a broad immune response, suggesting adjuvants are essential to vaccine design and development. Therefore, designing a potent adjuvant is key to vaccine development.

## 2. Alum

Alum has dominated all the adjuvants currently approved and licensed in the world despite a great deal of interest in developing novel adjuvants. This adjuvant was first used by Alexander T. Glenny who prepared potassium aluminum sulphate or alum {KAl(SO_4_)_2_}-adjuvanted vaccines by co-precipitation with Diphtheria Toxoid (DT) dissolved in carbonate buffer (Glenny and Sudmersen 1921; Glenny et al. 1926, 1931). Due to the problems in manufacturing reproducibility (Marrack et al. 2009), the technique of alum precipitation has been substituted by the adsorption of vaccines onto preformed aluminum hydroxide (AH) or alhydrogel (chemically crystalline aluminum oxyhydroxide) and aluminum phosphate (AP) or adju-phos gels (chemically amorphous aluminum hydroxyphosphate) (Baylor et al. 2002; Shirodkar et al. 1990). Aluminum compounds such as aluminum chloride, aluminum silicate, algammulin (gamma inulin plus AH), cesium alum (CA), Imject alum (IA) (AH plus magnesium hydroxide) have sometimes been substituted for AH and AP adjuvants in experimental studies (Lindblad 2004a, b; Lindblad and Schonberg 2010; Flach et al. 2011; Kool et al. 2008a, b; Marichal et al. 2011). Although these compounds are often generically referred to as ‘alum’, alum is in fact a distinct chemical composition (Hem et al. 2007). Importantly, few of these compounds have been used to address their comparative roles in the induction of immune responses *in vivo* (Cain et al. 2013).

While these adjuvants have been in continuous use in human vaccines for about 90 years, their mechanisms of action have remained elusive. A number of alum-induced effects may contribute to the improved immunogenicity of vaccines, however, in many cases these effects are only partially described or lack clear causal association with adjuvant function.

## 3. Mechanisms of action: *in vitro* vs *in vivo* paradigm

Adjuvant biologists have hypothesized that adjuvants work by ‘depot formation’, ‘Ag targeting’, and ‘inflammation’. These hypotheses are based on evidence from *in vitro* studies, with few *in vivo* validation studies. This is because the study of vaccine adjuvants remains largely empirical, despite our updated knowledge and understanding of immunology. Reductionist approaches, such as analyzing adjuvant effects on key immune system cells *in vitro* will help define the features of adjuvants that are critical for their function, and greatly enhance our understanding of the mechanisms involved. However, adjuvants ultimately have complex interactions with their environment at the interface of immunology, physiology, and anatomy *in vivo. In vitro* a single cell may exhibit different behaviors under different experimental conditions, therefore understanding how cells behave *in vivo* and what interactions they have with their environment will be crucial to fully understanding the mode of action of adjuvants. A number of reviews have been published relating to the mechanisms of action of alum (Gupta 1998; Gupta et al. 1995; Lindblad 2004a, b; Brewer 2006; De Gregorio et al. 2008; Marrack et al. 2009; Reed et al. 2009; Mbow et al. 2010; Hogenesch 2012; Awate et al. 2013; Reed et al. 2013; Kool et al. 2012), however, *in vitro* and *in vivo* data has never been fully compared and evaluated. Thus, this review compares *in vitro* and *in vivo* studies examining the mode of action of adjuvants.

### 3.1 Challenging the theory of ‘depot formation’

The depot hypothesis is the earliest proposed mechanism for adjuvant action, hypothesized by Glenny, Buttle and Stevens in 1931 after working on DT-precipitated in alum (Glenny et al. 1931). They excised a portion of skin containing the site of injection from guinea pigs 3 days after administration of alum-precipitated DT or soluble DT. They then homogenized the skin and injected the emulsion into naïve guinea-pigs. The alum-precipitated DT-recipients were successfully immunized whereas, the DT-recipients (controls) groups were not, as measured by anti-toxin titers. This experiment led them to generate a hypothesis that the slow elimination of alum-precipitated Ags over a long period of time from single injection site may enhance both primary and secondary stimulation resulting in the associated enhanced Ab titers (Glenny et al. 1931). Similarly, Harrison proved this hypothesis by transferring the alum nodules from one guinea pig into another guinea pig (Harrison 1935). White and colleagues suggested that depot causes persisting inflammation that stimulates immune cells within the regional LNs, and induction of local granuloma that recruit Ab-producing plasma cells (White et al. 1955). Consequent studies suggested that Ag was detected for 2 – 3 weeks in alumina gel-triggered granulomas (Osebold 1982). It was realized that strong adsorption to an adjuvant may ensure a high localized concentration of Ag for a period of time (Harrison 1935; White et al. 1955), that may be sufficient to allow Ag uptake and activation of APCs like DCs (HogenEsch 2002).

Hypothetically, depot theory might be explained on the basis of alum’s role in a strong binding strength with Ag. This results in retention of Ag at the injection site, and in slow release of Ag *in vivo*. Aluminum adjuvants have been shown to adsorb various proteins via either electrostatic interaction, ligand exchange, or via hydrophilic-hydrophobic interaction and each binding interaction depends on the nature of the Ag, pH, ionic strength, and presence of surfactants (Lindblad 2004a; Hem and HogenEsch 2007). As a result of adsorption, the soluble Ags change into particulate form (Lindblad 2004a, b). Therefore, compared with soluble Ags, particulates efficiently interact with APCs resulting in enhanced phagocytosis. As well as going through the internalization process, the soluble Ags may be trapped inside the irregular aggregates of large sized (1 – 10 μm) AH particles formed by fibrous primary particles (Powell et al. 1995). These primary particles in the aggregates are loosely associated and are readily degraded (Morefield et al. 2005) with the subsequent release of Ags in tissue culture media *in vitro* (Heimlich et al. 1999). The release is observed in similar manner *in vivo* because aluminum-containing adjuvants are rapidly chelated and solubilized by alpha-hydroxycarboxylic acids such as citric acid, lactic acid, and malic acid in the interstitial fluid, absorbed into the tissues, and finally eliminated in the urine (Hem 2002; Seeber et al. 1991). As release of Ags or elution of alum in media (*in vitro*) or interstitial fluid (*in vivo*) is a time-dependent phenomenon, initial Ag uptake by APCs may include adsorbed and trapped Ags in alum aggregates via phagocytosis. This is then followed by macropinocytosis of soluble protein released from alum aggregates (Romero Mendez et al. 2007). The elution of Ags from the adjuvant surface is crucial *in vivo* because residential LN APCs like DCs pinocytose the eluted Ags from the injection sites (Figure 1). Though macropinocytosis and presentation of Ag by residential LN DC is not sufficient to induce an immune response *in vivo* (Itano et al. 2003), a second wave of presentation of Ag by DCs originating from the injection site interact with naïve T cells in the DLN inducing an effective immune response. Thus, retention of Ag for phagocytosis by injection site DCs and sustained release of Ag for macropinocytosis by LN DCs are critical for the efficient Ag presentation in this situation (Lu and Hogenesch 2013). However, the retention of Ag and its slow release have been shown dispensable to alum adjuvanticity in the context of an enhanced Ab response *in vivo* (Romero Mendez et al. 2007; Noe et al. 2010; Flach et al. 2011). In these contexts, the adsorption and elution rates have been quantified using Ovalbumin (OVA) mixed in AH or Phosphate-treated AH (PTAH) (adsorption: 91%, complete elution in interstitial fluid: 4 hours), Dephosphorylated alpha casein (DPAC) mixed in AH (Adsorption: 100%; complete elution in interstitial fluid: <6 hour) or Phosphate-treated AH (PTAH-B) (Adsorption: 40%, complete elution in interstitial fluid: <1 hour) (Iyer et al. 2003). Considering these *in vitro* data*,* the authors injected these vaccine preparations subcutaneously in mice in which they observed an enhanced IgG titer *in vivo* independent of adsorption and elution characteristics of Ags (Iyer et al. 2003). It, therefore, suggests that strong adsorption might be detrimental to physical and chemical properties of vaccines (Estey et al. 2009; Vessely et al. 2009). Nevertheless a slight interaction between alum and Ag is necessary, for example, a favorable immune response has been observed after an interaction between alum and anthrax Ag (Watkinson et al. 2013) or hepatitis B Ag (Egan et al. 2009). The necessity for interaction between alum and Ag has been also proved by other experiments in which no immune response was generated following separate Ag/alum injections (Chang et al. 2001) though quantitatively higher Ab titer was obtained in one similar experiment (Flebbe and Braley-Mullen 1986). Therefore, the suggestion by the WHO recommending >80% of DT and tetanus toxoid (TT) Ags should be adsorbed (WHO 1977) and by the United States minimum requirements recommending >75% DT and TT Ags should be adsorbed (Anonymous 1956) onto aluminum adjuvants may not be right for other vaccine candidates. These are, however, *in vitro* recommendations and therefore have no bearing on what happens *in vivo*.

The Ag retention hypothesis, was not substantiated by subcutaneous (s.c.) immunization of mice with radioactively (^14^C)-labeled TT adsorbed to AP adjuvant (Gupta et al. 1996). Subsequently, a study was conducted to show the relation between Ag retention at the site of injection and Ab titers in rat sera 5 weeks after primary immunization and 2 weeks after boost (Noe et al. 2010). In this study, rats were injected with ^111^In-labelled alpha casein (IDCAS) Ag adsorbed to AH or IDCAS adsorbed to AP or non-adsorbed IDCAS Ag formulated in phosphate-treated AP (PTAP) or IDCAS solution by s.c. route. They observed Ag retention in the following order IDCAS + AH > IDCAS + AP > PTAP = IDCAS and the Ab titers in the order PTAP = IDCAS + AP > IDCAS + AH> > IDCAS suggesting an inverse correlation between retention and Ab titer (Noe et al. 2010). This study and that of de Veer and colleagues summarized that alum reduces the amount of soluble Ag entering afferent vessels, although their adjuvanticity was not found to be correlated with slow Ag release (de Veer et al. 2010; Noe et al. 2010). Thus, *in vivo* dispensability of strong adsorption, retention, and slow release of Ag by alum in Ab response indicates that other mechanisms may exist in alum adjuvanticity.

While the depot effect was considered as the predominant effect of alum, resulting in the sustained release of Ags over time (Cox and Coulter 1997), this role has been questioned since the 1950s (Holt 1950; Gupta RK 1996; Noe et al. 2010; de Veer et al. 2010; Hutchison et al. 2011). The depot effect was first challenged by Holt, who observed no change in Ab titers in response to DT following excision of the alum-precipitated injection site after 7 or more days (Holt 1950).

**Figure 2:**
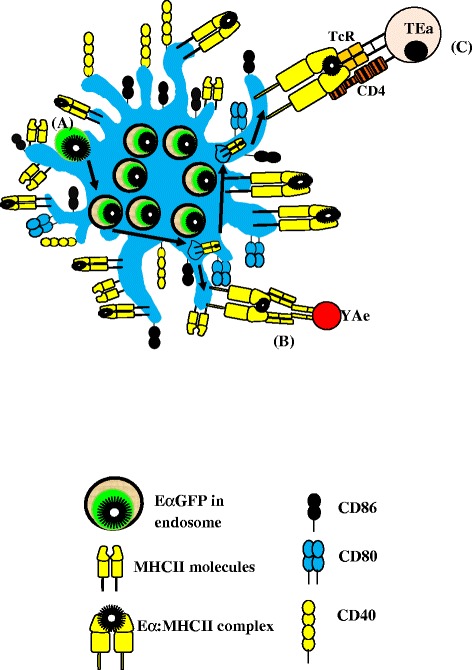
EαGFP:YAe system can be used to assess antigen (Ag) uptake, processing, and presentation by DCs in the presence of alum *in vitro* and *in vivo*. **(A)**: When EαGFP is taken up by DCs, it is processed and presented in the context of Eα(52–68):MHCII complexes. **(B)**: The Eα(52–68):MHCII complexes can be bound by YAe antibody (Ab). **(C)**: T cell receptor (TCR) of TEa mice sees the same complexes what YAe sees. Alum enhances Ag uptake, reduces degradation, and eventually increases presentation by DCs *in vitro*. This adjuvant also enhances the expression of CD86, CD80, and CD40 molecules by DCs *in vitro.* Arrow shows Ag processing path.

Recently, by using the E alpha green fluorescence protein (EαGFP)/YAe system, Hutchison and colleagues failed to observe activation of Ag-specific T cells transferred into recipient mice later than 5 days post-immunization with OVA + alum (Hutchison et al. 2011). When this Ag is internalized by DCs, EαGFP is degraded and the Eα peptide is presented by I-A^b^ MHC class II molecules on the cell surface. These p:MHCII complexes can be detected by staining the cells with YAeAb because this Ab can efficiently bind the complex of Eα(52–68) and I-A^b^MHCII (Rudensky et al. 1992; Rudensky et al. 1991; Rush and Brewer 2010; Ghimire et al. 2012). Therefore, the YAeAb recognizes the same epitope-MHC complex as the T cell receptor (Figure 2). This system allows assessment of Ag uptake/degradation and, in combination with the Y-AeAb Ag presentation *in situ* (Rush and Brewer 2010; Ghimire et al. 2012) (Figure 2). Notably, Hutchison’s studies further demonstrated a lack of Ag persistence and presentation in APC populations in the DLN (Hutchison et al. 2011). Remarkably, B cells were the first APCs to present Eα:MHCII complexes within 6–12 hours after immunization, then, cDCs presented these complexes within 12–24 hours following alum + EαGFP immunization, and pDCs presented Ags within 48–72 hours after alum administration in DLNs *in vivo* (Hutchison et al. 2011). No difference was observed in Ag uptake and presentation by B cells, cDCs, and pDCs after injection site ablation 2 hours following EαGFP administration, suggesting no role for the depot effect in alum adjuvanticity (Hutchison et al. 2011).

### 3.2 The alternative theory of ‘Ag targeting’

Landsteiner explained the mechanisms of action of alum in the context of slow absorption and delayed removal of Ag (White et al. 1955), suggesting that the ‘particulate’ nature of these adjuvants would favor phagocytosis by macrophages and subsequent activation. Further proof of Ag targeting by resident APCs was provided by the uptake of both Ags and AP by injection site macrophages following 7 days post-immunization of rabbits (White et al. 1955). This was a seminal study leading to the hypothesis of a mechanism of ‘Ag targeting’ for alum adjuvants. Ag targeting was defined in 3 distinct phases; the first phase involves the accumulation of cells at the injection sites, the second phase comprises of the induction of signal 1 and maturation signals by APCs, and the third phase includes the migration of Ag-loaded APCs into DLN.

#### 3.2.1 Alum induces chemokine- and cytokine- triggered APC recruitment at the injection site

Immunization with alum induces recruitment of various subsets of leukocytes such as neutrophils, eosinophils, macrophages, monocytes, and DCs at the injection site in a time-dependent manner (Mosca et al. 2008; Kool et al. 2008a; Lu and Hogenesch 2013) (Figure 1). This recruitment is associated with an enhanced expression of mRNAs for chemokines, cytokines, and cell adhesion molecules (Mosca et al. 2008; Kool et al. 2008a) and their secretions (Kool et al. 2008a; McKee et al. 2009; Korsholm et al. 2010), and enhanced production of complement cascade (Ramanathan et al. 1979). Various data on spatial and temporal recruitment of inflammatory cells has been acquired; this effect on cellular recruitment may be associated with the formulation of alum, Ag, and route of delivery. It has been recorded that following alum or alum + TT immunization, lymphoid tissue containing macrophages, epithelioid cells, and collagenous fibers is produced from 2 to 20 weeks (Goto and Akama 1982). Notably, neutrophils have been shown to migrate to the injection site at 6 hours (Lu and Hogenesch 2013), (Kool et al. 2008a), 24 hours (Calabro et al. 2011), and until 72 hours (Goto and Akama 1982) postimmunization by alum. Neutrophil trafficking is associated with increased expression of macrophage inflammatory protein 2 (MIP2) or chemokine (C-X-C) motif ligand 2 (CXCL2) and keratinocyte chemoattractant (KC) or CXCL1 (Kool et al. 2008a) that is enhanced via proteolytic cleavage of the extracellular matrix (Sadik et al. 2011). Depletion of neutrophils increased activation of Ag-specific T cell and the magnitude of the Ab response following s.c. immunization with AH + lysozyme (Yang et al. 2010), thus suggesting that neutrophils compete with DCs and macrophages for Ag and thus interfere with Ag presentation (Yang et al. 2010). In contrast, depletion of neutrophils with an anti-Ly6G Ab did not affect the magnitude and isotype of the Ab response to intramuscular (i.m.) immunization with AH + OVA (Lu and Hogenesch 2013). These different results are probably related to vaccine formulations and different route of immunization.

Macrophage trafficking to the injection site has been investigated for many years (White et al. 1955; Lu and Hogenesch 2013) with the peak of infiltration at day 7 (Lu and Hogenesch 2013) post alum immunization. The trafficking may be associated with an increased expression of CCL2 and CCL4 at the injection sites (Lu and Hogenesch 2013). Similar patterns of expression of chemokines by human monocytes have been reported *in vitro* in response to alum (Seubert et al. 2008) thus validating *in vivo* data. However, disappearance of macrophages from the alum-injected site has been shown in few studies (Kool et al. 2008a; McKee et al. 2009) and the different data may be associated with different protocol of immunization and adjuvant formulations. Importantly, the role of macrophages in alum adjuvanticity has been queried as systemic depletion of macrophages did not affect Ab response following intraperitoneal (i.p.) alum injection (McKee et al. 2009) and even enhanced the Ab response following s.c. alum immunization (Mitchell et al. 2012).

Similarly, the numbers of eosinophils increase (Calabro et al. 2011) within 24 hours (Kool et al. 2008a; Korsholm et al. 2010; McKee et al. 2008), and constitute about 25% of inflammatory cells when they peak around day 6 (Walls 1977; Lu and Hogenesch 2013) post alum injection. Eosinophil recruitment may be related to interleukin (IL)-5 and histamine release from mast cells, unidentified factors secreted by macrophages (McKee et al. 2009) and the secretion of eosinophil chemotactic protein or eotaxin-1 (CCL11) (Kool et al. 2008a) and CCL24 molecules following secondary immunization (Lu and Hogenesch 2013; McKee et al. 2009). Following immunization, mast cell numbers decreased at the site of injection probably due to mast cell granulation and cell death (McKee et al. 2009). Despite the fact that mast cells are the primary source of IL-5, IL-16, granulocyte-colony stimulating factor (G-CSF), KC, and MIP2 and that mast cells in concert with macrophages induce the secretion of IL-1β, IL-1 receptor antagonist (Ra), IL-6, and eotaxin; cell depletion studies suggest that these cells are not required for alum-mediated responses *in vivo* (McKee et al. 2009). The role of CCL11 is controversial with its receptor, CCR3, expressed on murine eosinophils, CCL11 has been shown to be dispensable in eosinophil recruitment following alum immunization (McKee et al. 2009). Despite eosinophils being the primary sources of IL-4 and possibly contributing to B cell priming and Ab production, their absence did not influence the quality or magnitude of the Ab responses to alum-adjuvanted vaccines *in vivo* (McKee et al. 2009).

Monocyte and DC trafficking at the injection site is particularly important as APC interactions are at the interface of the innate and adaptive immune response. The trafficking of monocytes is linked to the various signals derived from neutrophil (Soehnlein and Lindbom 2010). The notion that inflammatory monocytes can differentiate into DCs *en route* to the DLNs suggests that subsequent Ag-specific immune response occurs following immunization (Geissmann et al. 2003). It has been recorded that DCs are actively recruited to the injection sites from day 1 to day 7 postimmunization (Mosca et al. 2008; Kool et al. 2008a; McKee et al. 2009; Calabro et al. 2011; Lu and Hogenesch 2013). Depletion of DCs nearly completely abolished T cell responses and Ab production (Kool et al. 2008a) indicating DCs are critical for the alum-mediated enhanced immune responses *in vivo*. Recently, CD8α^+^ DCs have been considered to prime CD8^+^ T cells via cross-presentation following alum immunization. In this context, in absence of CD8α^+^ DCs, other unknown subsets of DCs have been shown in priming CD4^+^ T cells (MacLeod MK 2011).

#### 3.2.2 Alum enhances uptake and presentation of Ags by APCs

Alum adjuvants are reported to enhance the Ag uptake capacity of macrophages (White et al. 1955) and DCs (Kool et al. 2008a) *in vivo* and human peripheral blood mononuclear cells (PBMCs) (Mannhalter et al. 1985) and DCs (Morefield et al. 2005; Flach et al. 2011; Ghimire et al. 2012) *in vitro*. To illustrate alum’s role in enhancing Ag targeting efficiency of DCs *in vitro,* the EαGFP/YAe system has been used (Figure 2). This system has allowed the impact of AH on the intracellular Ag depot that provides a sustained release of Ag onto MHCII molecules and prolonged Ag presentation (Ghimire et al. 2012) to be assessed (Figure 2). Although the data is not validated by *in vivo* experiments (Hutchison et al. 2011), other factors such as DC’s potentiality to ingest alum particle and further consequences should be evaluated. *In vitro* experiment showed phagocytosis of alum particles by macrophages and consequently their phagosomal rupture (Hornung et al. 2008). Similar experiment by Flach and colleagues suggested that alum binds DC plasma membrane lipids in cholesterol- and cellular motility-dependent manner. Subsequently, the lipid sorting occurs resulting in enhanced Ag delivery to the cell without alum internalization, a process called abortive phagocytosis (Flach et al. 2011). In the same study, alum particles were identified inside DCs (Flach et al. 2011) and named them ‘confounding DCs’, indicating that DCs are efficient in phagocytic uptake of particulates. The *in vitro* experiments have been validated by *in vivo* observations, for example, alum crystals have been identified within s.c. injection-site granulomas by histochemistry, electron microscopy, X-ray microanalysis, and atomic absorption spectrophotometry (Frost et al. 1985; Miliauskas et al. 1993; Fawcett and Smith 1984). They have been identified inside macrophages (Rimaniol et al. 2004; Morefield et al. 2008), multinucleated giant cells (Morefield et al. 2008), and MHCII^+^ mononuclear cells, most probably the DC (Lu and Hogenesch 2013), and recently within a T helper 1 cell line (THP-1) (Mold et al. 2014) indicating these APCs actively engulf alum *in vivo*.

While influences on p:MHCII presentation by APCs in the presence of alum has been described *in vitro* (Ghimire et al. 2012) and *in vivo* (Hutchison S 2011), there is little data available on p:MHCI presentation. Understanding the effects on p:MHCI presentation is critically important particularly because alum has been reported to act as a poor cytotoxic T lymphocyte (CTL)-responding adjuvant which would be crucial for immune responses for intracellular pathogens like HIV, *Plasmodium*, and *Mycobacterium tuberculosis*. Importantly, one *in vitro* study of alum excluded class 1 processing and presentation in macrophages (Rimaniol et al. 2004). Few *in vivo* experiments have shown MHC class I-restricted CD8 T cell responses elicited by alum adjuvants. However, the induction of long-lasting protective CD8^+^ CTL response in mice primed and boosted with a highly purified recombinant influenza protein vaccine formulated in alum adjuvants has been reported (Dillon et al. 1992). The levels of CTL responses induced were similar to that obtained by an i.p. boost of Ag without alum adjuvant. Thus the vaccine containing alum induced CD8^+^ T cell response, but the alum itself did not necessarily contribute to the CD8^+^ T cell response (Dillon et al. 1992). This notion has been further clarified by subsequent experiments (McKee et al. 2009; MacLeod et al. 2011). McKee and colleagues observed both CD4^+^ and CD8^+^ T cell activation in the presence of alum (McKee et al. 2009). Ag (Kb/SIINFEKL tetramer)-specific CD8^+^ T cells and the consequently the development of long-lived memory cells have been uniquely shown *in vivo* following alum immunization (MacLeod et al. 2011). This study also addressed the generation of type 1 cytokine response and the consequent CTL function and the significant protection from influenza A challenge via alum-primed CD8^+^ T cells *in vivo* only in the presence of additional adjuvant called monophosphoryl lipid (MPL) (MacLeod et al. 2011). It further suggested that alum enhanced the generation of long-lived CD8^+^ T memory cells and MPL enhanced CTL differentiation indicating this type of vaccine formulation would be applicable to enhancing p:MHCII-primed Th1- and p:MHCI-primed-CD8^+^ T cell responses required for killing intracellular pathogens (MacLeod et al. 2011). It is therefore interesting to note that varying levels of adjuvanticity of alum have been reported *in vivo* depending on the protein or peptide used, and the addition of cytokines such as IL-12, and other TLR-agonists such as saponin (Newman et al. 1992), CpG and polyriboinosinicpolyribocytidylic acid (Chuai et al. 2013)*.* Importantly, the use of inflammatory cytokines such as IL-12 in alum has been shown to induce Th1 responses (Jankovic et al. 1997) with synergistic enhancement when coadministered with IL-18 (Pollock et al. 2003). Enhanced levels of interferon-gamma (IFN-λ) following immunization with *Bordetella pertussis* toxin mixed in alum (Toellner et al. 1998) were also reported. Interestingly, vaccination with recombinant hepatitis B serum Ag (HBsAg) adsorbed to AH induced the production of viral-specific CTL induction, Th1 induction, and Ab production (Rahman et al. 2000). Notably, the effects on CD4^+^ and CD8^+^ T cell response can be explained on the basis of respective upregulation of cluster of both MHCII genes (H2-Aa, H2-Ea, and H2-Eb1) and MHCI genes (H2-Q, H2-K, H2-T, H2-D) at the injection site by alum after 4 days and 1 – 2 days of post injection (Mosca et al. 2008) respectively. These experiments suggest that alum, as a particulate adjuvant (Cox and Coulter 1997), may have the potential to modulate processing of the protein Ags via the MHCI pathway for the CTL induction via cross presentation (Munks et al. 2010; Carbone and Bevan 1990). This may be possible if exogenous Ags are released into the cytosol from the endosomes by alternative mechanism, for example, by the release of Ags into the cytosol following damage to the phagosome as discussed later. This type of particulate delivery is also important in delivering both Ag and adjuvant into the same cell to enhance immune responses (Pashine et al. 2005; Bramwell and Perrie 2005).

#### 3.2.3 Alum enhances APC activation and maturation

Studies have suggested that aluminum-containing compounds have direct effects on DC activation as measured by expression of MHCII and costimulatory molecules (Figure 2). Ulanova *et al.* reported IL-4-dependent increased level of MHCII, CD86, CD83, IL-1α, IL-1β, tumor necrosis factor (TNF), IL-4, and IL-6 in human PBMCs which later acquired a DC morphology (Ulanova et al. 2001). Similarly, Rimaniol and colleagues showed that alum can trigger monocyte differentiation into myeloid DCs in an IL-4 dependent pathway (Rimaniol et al. 2004). Alum was shown to induce a dose dependent decrease CD80 expression and increase CD86 and CD40 expression in human PBMC-derived macrophages 48 hours post treatment (Rimaniol et al. 2004). This study also suggested that alum (AlOOH) can directly act on already differentiated macrophages and can change them to mature, specialized Ag presenting macrophage in an IL-4 independent manner (Rimaniol et al. 2004). In contrast to these studies, Sun and Brewer did not observe any significant increase in MHCII or costimulatory molecule expression on murine CD11c positive bone marrow-derived DCs (BMDCs) 24 hours post alum treatment (Sun et al. 2003). Sokolovska and colleagues in 2007 reported an increase in CD86 and CD80 expression on murine BMDCs (Sokolovska et al. 2007), while Seubert and colleagues observed an enhanced expression of CD86, MHCII, CD71, CD83, and CCR7 molecules and decreased expression of CD80 and CD1a molecules on DCs derived from the CD14-positive monocytes (Seubert et al. 2008). An enhanced CCL2, CCL3, CCL4, CXCL8, MHCII, CD86, CD71, CD54 and decreased CD14 molecule expression was also observed accompanied by the increased granularity of the monocytes derived from human PBMCs (Seubert et al. 2008). In this study a time-dependent increase in CD86 and decrease in CD80 molecules was noted on the surface of monocytes derived from human PBMCs. Flach and colleagues did not find any effect of CA (CA at 5 mg/mL concentration) on CD86, CD80, and CD40 expression on BMDCs derived from C57BL/6 mice 24 hours post treatment (Flach et al. 2011). The different patterns of costimulatory molecules induction by alum in these *in vitro* studies may be due to differences in experimental design, including variation in the dose of alum, duration of stimulation and host strains used. The differences in host strain is particularly important as the expression of maturation markers such as CD86, CD40, and Stat4, an IL-12-inducing gene, are quantitatively higher in spleen-derived DCs of C57BL/6 compared with BALB/c mice (Liu et al. 2002). Further *in vivo* studies should be conducted to address these issues in alum adjuvanticity.

#### 3.2.4 Alum aids recruitment of Ag loaded APCs to the lymphoid tissue *in vivo*

It is believed that following alum immunization, inflammatory Ly6C^+^CD11b^+^monocytes take up Ag, differentiate into CD11c^+^MHCIIDCs in a myeloid differentiation primary response gene 88 (MYD88)-dependent manner *in vivo* (Kool et al. 2008a) validating the *in vitro* differentiation of PBMCs into DC morphology (Rimaniol et al. 2004; Seubert et al. 2008; Ulanova et al. 2001). These DCs become long-lived after ingesting alum (Hamilton et al. 2000) and slow down alum solubilization (Verdier et al. 2005; Gherardi et al. 2001; Authier et al. 2006). These DCs subsequently gain enhanced inflammatory signals such as IL-1β, TNF-α, and IL-6 that induce their trafficking to DLN (Kool et al. 2008a). Nevertheless, the latter two cytokines have been shown dispensable for alum adjuvanticity (Brewer et al. 1998). Thus, IL-1β may principally play a role in DC mobilization to DLNs by decreasing E-cadherin mRNA expression in DCs resulting in the detachment of cells from neighboring cells and matrix components (Jakob and Udey 1998). In the LNs, DCs can transfer Ag material to a large network of distant LN APCs (Carbone et al. 2004) and can induce activation of innate immune system in distant organs (Wang et al. 2013). That is why alum has been reported to induce the immune responses in distant LN and spleen (Kool et al. 2008a).

Although isotopic ^26^Al-enriched alum was detected in LNs up to 28 days (Flarend et al. 1997), the kinetics of alum-fed APC migration and the fate of this adjuvant was explained by Khan and colleagues (Khan et al. 2013). Following s.c. or i.m. alum injection, alum-fed APCs were recruited into DLN from 1 hour to 21 days (peaking at 4 days) post alum administration, and into spleen from 1 hour to 90 days (peaking at 21 days) post alum administration in chemokine-dependent manner (Khan et al. 2013). The s.c. route of administration was superior to i.m. administration in recruiting alum-positive cells to the DLNs (Khan et al. 2013). Gr1^+^ cells are the major subsets that engulf alum particles, but the kinetics of alum-engulfed Gr1^+^ and Gr1^−^ recruitment to the DLN decreases with time. This phenomenon may be explained by dilution of particles by cell division (Kabashima et al. 2005) or particle transmission to other cells (Angeli et al. 2006).

Following migration to the DLN, alum-loaded DCs migrate to the bloodstream via thoracic duct and ultimately they can migrate distant organs such as spleen (Khan et al. 2013). In the spleen, DCs present Ags to the splenic T cells (Cavanagh et al. 2005). Interestingly, following 6 injections, increased accumulation of Gr1^+^/CD11b^+^ cells was noted in the spleens of alum-immunized mice, compared to the spleens of alum adsorbed to protein-immunized mice (HBsAg-anti-HBs), suggesting alum alone triggers effective inflammatory responses *in vivo* (Wang et al. 2013).

#### 3.2.4 Alum generates a strong default Th2 response

*In vitro* studies have shown that alum potentially induces the production of IL-1β and IL-18 from DCs and the consequent expansion and differentiation of naïve CD4^+^ T cells into Th2 cells and promote the production of Abs (Sokolovska et al. 2007). It has been experimentally shown that IL-4 and IL-13 are not necessary for alum to enhance Th2 responses but these cytokines strongly suppress Th1 responses, thus alum’s role in regulating Th2 response may be mediated via Th1 suppression (Brewer et al. 1996; Brewer et al. 1999). Although IL-4 has been shown to direct both CD4^+^ and CD8^+^ T cells to produce Th2 cytokines *in vitro*, only CD4^+^ T cells produce this cytokine in alum-mediated response *in vivo* (Serre et al. 2010). Alum’s induction and enhancement of Th2 responses has been validated by *in vivo* experiments. Alum-adjuvanted Ag contact with CD4^+^ T cells promotes migration to the B cell follicles and the development of a central memory CD4^+^ T cells. These CD4^+^ T cells undergo expansion and migrate from DLN and to distant LNs not exposed to Ag (Luther et al. 2007; Serre et al. 2009). In the distant LNs, these CD4^+^ T memory cells migrate to B cell follicles and produce IL-4 for the Th2 responses (Luther et al. 2007; Serre et al. 2009). Alum has also been suggested to enhance Ab responses via various molecules such as IL-4 (Grun and Maurer 1989; Jordan et al. 2004; Serre et al. 2011; Cunningham et al. 2004a; Pai et al. 2004; Zhu et al. 2010; Lindblad et al. 1997), IL-1 (Grun and Maurer 1989), IL-25 (Serre et al. 2008), IL-6 (Serre et al. 2008), IL-10 (Lindblad et al. 1997), IL-13 (Serre et al. 2011), GATA-3 molecules (Cunningham et al. 2004b; Pai et al. 2004; Zhu et al. 2010) and CXCR5 chemokines (Serre et al. 2011). These molecules probably trigger induction of Ab-forming cells, germinal center B cell responses through inflammation (Cain et al. 2013). Interestingly, induction of Th2 cytokines *in vivo* in absence of IL-4 or STAT6 signaling (Cunningham et al. 2002; Cunningham et al. 2004a, b; van Panhuys et al. 2008) suggests that an alternative pathway of *in vivo* Th2 induction exists in alum adjuvanticity. An enhanced Ab response has been addressed in detail *in vivo* by Zlatkovic and colleagues (Zlatkovic et al. 2013). They hypothesized that immunization of mice with alum adjuvanted inactivated purified flavivirus tick-borne encephalitis particle leads to the fixation of the flexible protein subunits in a certain configuration because alum present within the LN may mask certain epitopes of incompletely desorbed Ag that results in an enhanced and high-specific and high-avidity Ab response (Zlatkovic et al. 2013).

### 3.3 The theory of ‘inflammation’

The use of adjuvants in vaccination is usually associated with some degree of injection site inflammation, and this process is considered an essential part of adjuvant function (Qin et al. 2009). This is consistent with the ‘Danger Theory’ of immune activation as proposed by Polly Matzinger in 1994 (Matzinger 1994). According to this theory, initiation of the immune response is not dependent on microbial recognition, but rather on the ability of pathogens or other agents such as adjuvants to cause tissue damage. The danger signals released from damaged tissues then have the capacity to drive inflammation and initiate an adaptive immune response (Matzinger 1994). This provides an important mechanistic theory for alum adjuvants with many reports of inflammatory effects at the injection site and the induction of danger signals from cells following alum interaction. For example, nodule or granuloma formation in humans and animals following alum injection has been reported from the 1930s to present day (Glenny 1931; Harrison 1935; Farago 1940; Holt 1950; White et al. 1955; Munks et al. 2010; Lu and Hogenesch 2013; Vogelbruch et al. 2000; Bordet et al. 2001; Chong et al. 2006; Rock et al. 2010; Marsee et al. 2008). The development of alum granuloma is independent of the route of immunization and occurs from a few days to several years (e.g., up to >12 years) following immunization, supporting the hypothesis that vaccines containing alum lead to a short-term inflammatory effect in a normal environment as well as long-term inflammatory effects in a pathological environment, at the site of injection (Gherardi et al. 2001; Kool et al. 2008a). It has been shown that alum induces uric acid or monosodium urate (MSU) crystal as a danger signal (Kool et al. 2008a). Subsequently, other signals such as heat shock protein 70 (HSP70) (Wang et al. 2012), and deoxyribonucleic acid (DNA) (Marichal et al. 2011; McKee et al. 2013) have been illustrated as inducers of alum-mediated immune responses, indicating that the mechanisms by which alum particles induce inflammation is central to understanding its adjuvant properties.

AH is able to induce caspase-1 activation and trigger the release of IL-1β and IL-18 by both human and mouse DCs via caspase-1 activation in a MyD88-independent fashion *in vitro* (Li et al. 2007).The use of BM mononuclear cells from NLRP3^−^/^−^ and wildtype mice demonstrated lipopolysaccharide (LPS) is not required for the alum (AH, IA)-mediated caspase-1 activation via NLRP3 signaling *in vitro*, however, LPS is necessary for pro-ILβ expression (Li et al. 2008). The experiments showed that caspase-1 activation and processing and secretion of IL-1β in response to alum is mediated by the NLRP3-inflammasome in both mouse and THP-1 cell line confirming the role of NLRP3 in alum adjuvanticity (Li et al. 2008). By taking wild type and LPS-primed NLRP3^−^/^−^or apoptosis-associated speck-like protein containing C-terminal caspase-recruitment domain knockout (ASC^−^/^−^) macrophages, it has been shown that alum (AH) mediates IL-1β secretion via NLRP3 inflammasome in an adenosine triphosphate (ATP)-independent manner *in vitro* (Franchi and Nunez 2008). It has been suggested that alum alone is insufficient to trigger caspase-1 activation because it requires priming or costimulation with LPS. However, i.p. immunization of NLRP3^−^/^−^ mice with human serum albumin (HSA, a T cell dependent Ag) and alum did not reduce the significant production of anti-HSA Ab *in vivo* (Franchi and Nunez 2008). Likewise McKee and colleagues, using DT or alum (AH), proved that the absence of caspase-1 or NLRP3 was not associated with altered CD4^+^ or CD8^+^ T cell responses, Th2 induction or Ag-specific IgG1 production *in vivo* (McKee et al. 2009). In contrast to these studies, it was shown that Ag-specific IgG1 and IL-5 production was reduced following immunization of NLRP3-deficient mice with OVA adsorbed to alum (IA) or HSA adsorbed to alum (Eisenbarth et al. 2008) indicating alum adjuvanticity is dependent on the NLRP3 pathway. This study illustrated a reduced innate immune response, specifically reduced airway eosinophilia, in the absence of NALP3 signalling, indicating a significant potential contribution to the adjuvanticity of alum (Eisenbarth et al. 2008). Similarly, *in vitro* studies using macrophages from NALP3^−^/^−^ and ASC^−^/^−^ mice suggest that the NALP3 molecule is required in alum-mediated caspase-1-triggered pro-IL-1β processing (Kool et al. 2008b). *In vivo* studies using NALP3^−^/^−^ mice suggest that alum activates DCs, increasing the expression of CD86 and MHCII molecules, and their trafficking to DLN in an IL-1β dependent manner, resulting in increased T cell division, and also increases the levels of OVA-specific IgE and IgG2c. Studies have also shown that alum activates caspase-1 in MyD88^−/−^, indicating no role of TLRs in alum-mediated inflammasome activation *in vitro* (Kool et al. 2008b). In contrast, the same groups have recorded that MyD88 molecules are necessary for the alum-mediated recruitment of inflammatory monocytes to the DLN *in vivo* (Kool et al. 2008a). *In vitro* studies using uricase to neutralize the alum-released uric acid resulted in only a minor effect on IL-1β processing suggesting no key role for this molecule in alum-mediated NALP3 activation (Kool et al. 2008b). However, an *in vivo* murine model using uricase to neutralize uric acid prior to alum immunization demonstrated reduced inflammatory monocyte trafficking to the DLN and consequently reduced T cell division (Kool et al. 2008a). This, in contrast to *in vitro* study, suggests that releasing uric acid acts a danger signal and contributes alum (IA) adjuvanticity (Kool et al. 2008a). Notably, the difference in the results in these labs may be associated with the differences in experimental design, such as the genetic background of the mice used, formulations of alum used, different routes of immunization, contamination with TLR agonists, and incomplete characterization of the alum induced immune response (De Gregorio et al. 2008; Lambrecht et al. 2009).

**Figure 3:**
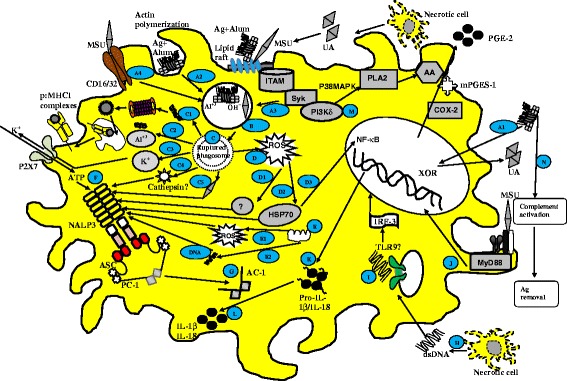
Hypotheses of NALP3 activation via inflammatory pathway. **(A1)**: Alum activates XOR and induces the secretion of uric acid (UA). **(A2)**: Alum-adsorbed antigen (Ag) is internalized by DC via actin-polymerization pathway. **(A3)**: Alum interacts with lipid raft of DC membrane and activates ITAM-Syk pathway to internalize Ag. **(A4)**: Alum-induced production of monosodium urate (MSU) crystals may be internalized by DC in CD16/32-dependent pathway. **(B)**: Alum in phagosome enhances maturation into phagolysosome and release of Al^3+^ and OH^−^. **(C)**: Al^3+^ inhibits the cathepsin L activity and consequently the generated stress induces the rupture of phagolysosomes. **(C1)**: The leaked Ag may bind with MHCI molecule in cytoplasm. **(C2)**: Nanometer-sized Al^3+^ ion precipitates in the cytosol. **(C3)**: The released K^+^ will be exported outside and extracellular adenosine tri-phosphate (ATP) will enter inside via P2X7 molecule. **(C4)**: Cathepsin molecule will be released in the cytosol. **(C5)**: MSU is released in cytosol. **(D)**: Reactive oxygen species (ROS) is generated. The ROS will activate various molecules such as **(D1)** unknown or **(D2)** Heat shock protein (HSP)70 that can trigger nuclear factor kappa-light-chain-enhancer of activated B cells (NF-κB) signaling and induces the secretion of IL-1β **(D3)**. **(E)**: Mitochondrion becomes activated and generates ROS **(E1)** and mitochondrial deoxyribonucleic acid (DNA) **(E2)**. **(F)**: **C3**, **C4**, **C5**, **D1**, **D2**, **E1**, or **E2** may activate nucleotide-binding oligomerization domain family-like receptor pyrin domain containing 3 (NALP3) and consequently AC-1 **(G)**. (**H):** Necrosis-induced release of double stranded (ds)DNA enters cytoplasm and initiates toll-like receptor (TLR)9–interferon regulatory factor (IRF)-3 signaling **(I)**. **(J)**: MSU molecules directly activate myeloid differentiation (MYD)88 signaling. **(K)**: Pro-IL-1β and IL-18 may be transcripted from nucleus. **(L)**: Active caspase (AC)-1 induces the secretion of their matured forms. **(M)**: immunoreceptor tyrosine-based activation motif (ITAM)-Syk-PI3Kδ pathway activates secretion of prostaglandin E2 (PGE2). **(N)**: Alum activates complement.

Recently, it has been shown that Ag-specific IgE is reduced in prostaglandin E2 (PGE2) synthase-deficient mice and that alum induces the production of the PGE2 by the Syk and p38MAP kinase pathway in an inflammasome-independent fashion (Kuroda et al. 2011). In this context, the immunoreceptor tyrosine-based activation motif (ITAM)-syk-phosphoinositide 3-kinase delta (PI3Kδ) pathway activates cytosolic phospholipase A2 (cPLA2) via p38 mitogen-activated protein kinase (MAPK) that results in the release of arachidonic acid (AA) from membrane lipids. AA is then converted into PGE2 by cyclooxygenases (COX)-2 and microsomal prostaglandin E synthase (mPGES)-1 molecule. The secreted PGE2 has the capability to trigger Th2 responses (Figure 3).

#### 3.3.1 Molecular mechanism of alum particles on inflammation

Inflammation is a component of the innate immune response that is comprised many proteins such as of nucleotide-binding oligomerization domain family-like receptor pyrin domain containing 3 or NLR protein 3 (NLRP3 or NALP3) inflammasome of macrophages and DCs (Martinon et al. 2007; Schroder and Tschopp 2010; Martinon et al. 2009). NALP3 consists of a domain present in neuronal apoptosis inhibitor protein (NAIP); MHC class II transactivator (CIITA), incompatibility locus protein from *Podospora anserine* (HET-E) and telomerase-associated protein (NACHT domain), and NACHT-associated domain (NAD) in between amino-terminus pyrin domain (PYD) and C-terminus leucine-rich repeat (LRR) (Schroder and Tschopp 2010). Notably, all the damage signals (Figure 3) activate the PYD of NALP3 that subsequently binds with the PYD of ASC. Consequently, the C-terminal caspase-recruitment domain (CARD) of ASC binds with CARD of pro-form of caspase-1, apoptosis-related cysteine peptidase (CASP1) domain that undergoes autocleavage at p20 or p10 sites. This results in the formation of the active caspase-1 p10/20 tetramer that processes the pro-forms of cytokines such as IL-1β into mature, active conformations and induces their secretion via unknown pathway (Agostini et al. 2004; Dinarello 2010; Schroder and Tschopp 2010). Similar mechanisms may occur during maturation and secretion of IL-18 (Martinon et al. 2009; Martinon et al. 2007). In contrast to the maturation process of IL-1β and IL-18, that of IL-33 is different. Although the secretion of mature IL-33 was triggered by alum treatment in THP-1 cells, and depended on ASC and NLRP3 (Li et al. 2008), caspase-1 processing actually causes inactivation of IL-33, rather than its activation (Cayrol and Girard 2009). The processing of caspase-1 is less effective in cleaving IL-33 compared with that of apoptosis-associated caspases (AAC) such as caspase-3 and caspase-7. However, the AAC-mediated IL-33 proteolysis has capacity to reduce IL-33 biological activity *in vitro* and *in vivo* (Luthi et al. 2009). Alum therefore induces the release of biologically active IL-33 as an endogenous danger signal via necrosis. In addition, it induces cleaving of IL-33 leading inactivation of its pro-inflammatory properties via apoptosis.

Alum’s role in the inflammasome (NLRP3) activation and inflammation is first mediated by enhanced phagocytosis (Hornung et al. 2008) followed by lysosomal acidification, resulting in targeting and activation of proteolytic enzymes found in lysosomes (Re 2011) (Figure 3). Consequently this induces stress (Pollock et al. 1995) leading to increased levels of the reactive oxygen species (ROS) such as the superoxide anion (O_2_^**.**-^) and hydrogen peroxide (H_2_O_2_), resulting in the rupture of the lysosome. Rupture of the lysosomal compartment by alum or crystals has been shown to be sufficient to activate the NALP3 molecule (Hornung et al. 2008).

The generation of O_2_^**.**-^ and H_2_O_2_ occurs via phagocytic nicotinamide adenine dinucleotide phosphate (NADPH) oxidase cytochrome b system (Martinon 2010; Holmstrom and Finkel 2014). Notably, the impact of alum and other particulates on phagocytic NADPH system has been studied in detail (Hornung et al. 2008). The importance of NADPH has been demonstrated in murine models, with mice deficient in this enzyme failing to produce phagocytic O_2_^**.**-^ and becoming hypersuceptible to various pathogens (Pollock et al. 1995). It has also been found that ROS triggers dissociation of thioredoxin interacting protein (TXNIP) that interacts with NLRP3 molecule from thioredoxin (Zhou et al. 2010). TXNIP deficient mice have also been shown to lack NLRP3 inflammasome activation and consequently lack of IL-1β secretion (Zhou et al. 2010). In contrast to this result, macrophages obtained from the mice lacking gp91phox, a phagosomal NADPH cytochrome b, retain activation of the NALP3 inflammasome in response to silica crystals, MSU, ATP, and poly (dA:dT) (Hornung et al. 2008). This suggests that the phagosomal respiratory-burst oxidase system is not necessarily involved in NALP3 activation (Hornung et al. 2008). This evidence is supported by the fact that the zymosan, a TLR2 agonist did not induce activation of the inflammasome in macrophages in spite of its potent phagocytic and ROS inducing properties (Hise et al. 2009; Joly et al. 2009). Thus, the notion that ROS may generate a specific but unknown ligand that can either activate NLRP3 or can modify this inflammasome or associated proteins (Dostert et al. 2009; Dostert et al. 2008; Martinon 2010) is being questioned.

In the same way, following lysosomal breakdown, HSP70 and K^+^, cathepsin molecules and MSU are released in the cytosol (Holmstrom and Finkel 2014; Pollock et al. 1995; Hornung et al. 2008). Alum has been shown to generates stress-triggered HSP70 molecules in CD11c^+^ DCs (Wang et al. 2012). Alum induced the cell surface and intracellular expression of HSP70 that activates either NF-κB pathway or NLRP3 inflammasomes (Wang et al. 2012). HSP70, an important molecular target for vaccines due to its role in enhancing Ag uptake and presentation by DCs (Basu et al. 2001), has been shown to enhance innate and adaptive immune response *in vivo* (Wang et al. 2012). This role has been confirmed in a murine model immunizing BALB/c with the mixture of alum (AH) in the presence of phenylethyne sulphonamide, an inhibitor of HSP70 (Wang et al. 2012).

Cathepsin B enzyme has also been shown to be released in the cytosol from the lysosome *in vitro* (Hornung et al. 2008). This enzyme causes pyroptosis, the NLRP3-mediated cell death that is inhibited when a cathepsin B inhibitor is used (Hornung et al. 2008). This suggests that cathepsin B is involved in NLRP3 activation. However, inflammasome activation was not affected in cathepsin B-deficient mice (Dostert et al. 2008). It is therefore possible that alum may contribute via other cathepsin molecules still present in cathepsin B-deficient mice (Felbor et al. 2002).

Following alum internalization, cellular perforation occurs resulting in the efflux of the potassium ion concentration (K^+^) channel via P2X7-pannexin-1 signaling and the consequent caspase-1 activation (Martinon 2010; Kanneganti et al. 2007). However, P2X7 and pannexin have been shown to be redundant in NLRP3 activation by MSU or alum (Re 2011), indicating the selective role of K^+^ efflux in inflammation.

In parallel to the effects on the phagosome, *in vitro* experiment using THP-1 showed increased mitochondrial activity following alum treatment, without any disruption to the mitochondria (Ohlsson et al. 2013). This has questioned the generally accepted theory that mitochondrial dysfunction may occur, releasing danger signals, such as oxidized mitochondrial DNA and ROS, into the cytosol (Nakahira et al. 2011; Shimada et al. 2012). This danger signal theory had been generalized as the secretion of inflammatory cytokines and mitochondrial ROS in NLRP3^−/−^ and ASC^−/−^ cells were reduced, indicating that mitochondria can sense intracellular stress and promote NLRP3 activation in a ROS-dependent manner (Zhou et al. 2011). *In vitro* model containing primary phagocytic cells such as DCs and macrophages rather than cell lines such as THP-1 may have more physiological relevant readouts and thus may be more informative in unraveling the mechanisms of action of alum.

#### 3.3.2 Induction of immune responses via the release of DNA and induction of complement molecules

*In vitro* studies have shown that AH enhances immature and murine bone marrow-derived macrophage (BMDM) survival and induces DNA synthesis, and enhances proliferative response to granulocyte macrophage colony stimulating factor (GMCSF) and colony stimulating factor (CSF)-1 (Hamilton et al. 2000). In contrast to this study, alum has also been shown to trigger necrosis of cells resident at the alum-injection sites *in vivo* (McKee et al. 2013; Cain et al. 2013; Marichal et al. 2011). The released DNA has been shown to trigger the TLR9-interferon regulatory factor (IRF)3-dependent pathway, activate inflammatory DCs, and enhance Th2 responses (Figure 3). In addition, the DNA has potentiality to activate IRF3-independent pathways, activate Tfh responses, and enhance B cell responses accompanied by IgE isotype switching and IgG1 production (Marichal et al. 2011). Although Marichal *et al*., demonstrated reduced DC migration from the peritoneal cavity to the DLN following deoxyribonuclease (DNase) treatment and i.p. alum injection, in a murine model, McKee *et al*., reported no effect on accumulation of, or expression of costimulatory proteins on Ag-loaded DCs in DLNs of stimulator of interferon genes (STING) mice following i.m. immunization (McKee et al. 2013). The latter groups found that DNase inhibited prolonged Ag presentation and DC-T cell interactions after i.m. injection in these mice. Thus demonstrating that host DNA introduced into the cytoplasm with the activation of STING pathway of Ag-bearing DCs enhanced p:MHCII presentation and DC-T cell interactions (McKee et al. 2013). Other factors such as histone proteins may be involved in alum mediated innate immune responses (Munks et al. 2010). Following i.p. or i.m. immunization, alum accumulates into nodules that are held together by host chromatin, released from inflammatory cells (Munks et al. 2010). The DNA released in this deposited chromatin may contribute to the adjuvanticity of alum *in vivo*.

AH has been shown to activate complement via the lectin pathway that results in the production of anaphylatoxins and complement component (C) 3b (Ramanathan et al. 1979). This effect is dependent on plaminogen for C3 activation. Importantly, alum binds complement or prothrombin incubated with plasma depending on the incubation temperature (Polley and Nachman 1975). The surface of aluminum has been shown to bind C3 (Arvidsson et al. 2007). However, complement deposition on AH has not been detected in a study (Tengvall et al. 1998). The role of complement *in vitro* has been validated by an *in vivo* experiment in which complement receptor-deficient mice had an impaired response to alum adsorbed to Ag injection (Chen et al. 2000). *In vitro* data has shown that AH activates the lectin pathway, alternative pathway, and classical pathway; however, it has the greatest effect on the alternative complement pathway. The alum provides a surface for complement activation, Ag opsonization, and Ag can be released via interactions with complement receptors (Guven et al. 2013) (Figure 3).

## 4. Conclusions and future directions

Aluminum compounds are the only adjuvants widely approved for use in human vaccines due to their safety record, ease of preparation, stability and immunostimulatory effects (Tritto et al. 2009; O'Hagan and De Gregorio 2009; Mbow et al. 2010). Applications include incorporation in vaccines that are successfully used against human papillomavirus (AH in Cervarix) and hepatitis B virus (AP in Fendrix), indicating the significant contribution of alum in mixed adjuvant preparation. Adjuvant development is undoubtedly a long process that must address regulatory, safety, and economic concerns, during clinical and preclinical development. Therefore, despite our incomplete understanding of the mode of action of aluminum based adjuvants, in the absence of suitable alternative adjuvants, alum is the sole candidate for use in human vaccines in the future. Considering the widespread use of alum, further comparative *in vitro* and *in vivo* should be undertaken to establish the mode of action due to the conflicting *in vitro* and *in vivo* data published. It is difficult to translate the *in vitro* data to the dynamic immune system essential for vaccine development, thus physico-chemical characteristics established *in vitro* and *in vivo* data regarding its role in inflammation can be exploited for future vaccine development. A clear causal association of alum in triggering immune responses via cellular death and or enhancing the quality, duration, and magnitude of T- and B- cell responses will make a significant contribution to the rational design of effective and safe vaccines and development of new adjuvants for future use.
